# Applying sequences of combinations of movements based on motor development stages principles to elite athletes: conceptual foundations and testable hypothese

**DOI:** 10.3389/fspor.2026.1755089

**Published:** 2026-04-30

**Authors:** Hamidou Msaidie, Danielle Gomez-Merino, Mounir Chennaoui

**Affiliations:** 1 Laboratoire de Biologie de L’Exercice Pour la Performance et la Sante (LBEPS), Université Paris-Saclay, Evry, France; 2 SLOWMOTIONS®, Madrid, Spain; 3 Université Paris Cité, URP7330 VIFASOM (Vigilance Fatigue et Sommeil), Paris, France; 4 Institut de Recherche Biomédicale des Armées (IRBA), Brétigny sur Orge, France

**Keywords:** body, brain, motor skills, movements, neuroplasticity, elite professional athletes

## Abstract

**Objectives:**

This article proposes a specific program of sequences of combinations of movements (SCM) based on motor development stages (MDS) to optimize the sports performance of elite professional athletes (EPAs).

**Design:**

Narrative literature review.

**Method:**

Information on MDS, including biomechanical, physiological, psychological skills, prevention, recovery, and training programs of EPAs, was extracted from 57 conceptually relevant references. Databases (PubMed, Scopus, Web of Science, Google Scholar) were searched using terms such as motor development, neuroplasticity, sensorimotor coordination, and sports performance.

**Background:**

The literature shows consistent evidence supporting the relevance of motor development stage–derived movement patterns for athletic performance. MDS are described as sequential postural and movement patterns emerging during early neuromotor maturation. They are widely applied in neurological rehabilitation, where studies report improvements in motor control and coordination, although transfer to elite athletes remains untested. Performance-related dimensions (posture, balance, mobility, coordination) are linked to central nervous system organization and neuroplasticity. Structured developmental movement sequences may enhance sensorimotor integration and motor refinement. Biomechanical and neurocognitive evidence suggests that SCM based on MDS could improve movement quality, neuromuscular efficiency, and adaptability. Some studies also associate motor learning with psychological factors such as attention and resilience, though evidence remains indirect. Overall, SCM appears as a promising multidimensional training approach, but lacks direct experimental validation in elite sport.

**Conclusions:**

With SCM, coaches, trainers, and scientists could design training and competition programs tailored to athletes’ individual needs, including warm-up routines, injury prevention, recovery, and adaptation to intense training and competitive situations. However, studies examining the acute and chronic effects of SCM on the biomechanical, physiological, and psychological responses of EPAs, including neuroplasticity, are needed to confirm this hypothesis and clarify its potential benefits across athletic domains.

## 1 Introduction

The Motor Development Stages (MDS)—in French, Niveaux d’Évolution Motrice (NEM)—described by Michel Le Métayer are applied during the rehabilitation of children with neurological disorders with sensory and motor dysfunctions (1–3). Whether congenital or acquired, these include various neuropathologies requiring rehabilitation, such as cerebral palsy and different types of paralysis, including hemiplegia, paraplegia, and quadriplegia (4–6). Signs and symptoms of neurological disorders include often sensory, motor, functional and cognitive consequences, and limitations in activities of daily living (ADLs) and quality of life (QoL) (7–9). Thus, the neurological rehabilitation has been the subject of numerous theories, control models, and motor learning activities over time (8, 10).

Rehabilitation based on MDS aims to acquire the main functions: (i) postural function, linked to the organization of movement; (ii) anti-gravity function, responsible for support, maintenance and straightening; (iii) locomotion function, integrating automatic walking, crawling and turning. It involves 17 steps caracteristics of the different phases of postures and changes in position that infants perform, from birth and throughout their motor development, to go from lying on their back (supine) to standing for walking (1, 2) (Table 1). The study of Vaubourg et al. (3) evaluated the efficacy of MDS in hemiplegic patients for walk rehabilitation, and the study of Cabanas-Valdés et al. (11) showed the contribution of MDS-inspired exercises to improve walking skills, following particularly the execution of core exercises performed in intermediate positions such as supine or seated position. In fact, techniques or rehabilitation exercises inspired by MDS, seem to allow an improvement in walking skills, regardless of the time and the theoretical justifications (1–3) (Table 2).

**Table 1:** The 17 steps of the MDS sequences as described by Le Métayer (1).

17 steps of the MDS sequences
1. Supine
2. Prone position: position of the sphinx
3. Turnovers: back to stomach and stomach to back
4. Crawl
5. Rabbit position (kneeling): sitting on its buttocks and held by its hands
6. On his knees erect: without support of the hands
7. Sitting beach position (position of the little mermaid): from the rabbit position, sliding the pelvis to the side
8. Sitting stable
9. Position of the serving knight
10. Squatting position: with support of the hands, then without support
11. Bear position: resting on his hands and feet, the child lifts his pelvis upwards
12. All fours position, ready to crawl in all fours position
13. Passage to a standing position: often from the position of the serving knight
14. Standing with support of both hands
15. Stand with one-hand support
16. Standing without support
17. Walk

**Table 2:** The benefits of MDS in pediatric and adult cerebral injury patients.

Benefits of MDS in pediatric and adult cerebral injury patients	
Articular	Improved mobility
Muscular	Tone: decreased in cases of spastic muscles; increased in cases of flaccid muscles; Elasticity: found
Sensitive	Perception and proprioception: restored
Motor	Motor control: developed and perfected
Functional	Changes of position, transfers, ground perception, walking; facilities
Activities of Daily Living (ADL)	Perfected
Quality of life (QoL)	Enhanced
Patterns of innate postures and movements	Balance, coordination; tweaked

The neuro-psycho-motor development is an essential criterion for reflection and research in favor of an evolution of rehabilitation in the field of neurology, and many authors have participated in research on motor development, in particular that of children. Jean Piaget, a Swiss psychologist, creates in 1923, the constructivist approach which considers the development and evolution of the child through a constructive process consisting of successive stages (12). Between 1930 and 1970, the stages of psychomotor development in children have been described (13–15), and universally consider that children go through a series of stages (“motor milestones”) between supine position, standing, then walking. These stages are directly linked to brain maturation, itself determined genetically (16). As early as 1946, Pickler, a Hungarian pediatrician, described functional levels of mental organization centered on motor, cognitive, affective and social aspects, and developed the idea that it is not necessary to teach to a baby how to walk (17). The pediatrician Michèle Forestier (18), a contemporary French physiotherapist, combines three phases in the journey towards safe and independent walking: the discovery of the body, space and verticality. According to Konkel (19), the cerebral maturation completed at twenty-five years, originates from the twenty-fifth week of pregnancy. These studies seem to confirm the interest of MDS expressed by Le Métayer in rehabilitating patients with neurological disorders, to restore sensory, motor and functional disorders, as well as ADLs, and QoL, through patterns of innate postures and movements.

Although rehabilitation and elite sport differ in goals and populations, they both rely on shared sensorimotor principles such as motor learning, neural plasticity, proprioceptive integration, and adaptive coordination. These shared mechanisms justify exploring developmental movement frameworks in performance contexts. Elite sport environments impose constraints distinct from rehabilitation contexts, including time pressure, high mechanical loads, multitask decision making, and competition stress. Although existing warm-up and neuromuscular programs reduce specific injury risks, they often target isolated components rather than integrated neurosensorimotor coordination. This highlights the potential value of complementary frameworks based on developmental motor organization.

In the high-level sport circle, EPAs train a lot in order to compete. The training sessions serve to repeat patterns, jumps, sprint races, long runs. They have to manage with functional challenges as simple as to walk, to fall down and to stand up correctly and very quickly. We know that elite sportsmen work on skills in high level performance, the execution of precise movements at low, medium and high intensity, and cognitive abilities, such as attention and decision-making, in order to improve, predict and achieve a high-performance result (20–23). Moreover, the efficiency of the warm-up routine is fundamental to accomplish the expected gesture, and to prevent injuries, as indicated in team sports (23–27). To this end, many programs have been created, particularly in soccer, like the FIFA 11+ Kids (28–30); and still other studies propose efficient solutions in terms of prevention, rehabilitation and neuro-training (31, 32). In the athlete, mobility, flexibility, strength, speed, coordination, spatio-temporal orientation, proprioception, neuromotor accuracy, analytical capacities are crucial (33–36). Deficits and disorders in these variables can translate into lack of precision (37–39). In addition, it is known that, psychological issues, fatigue, stress, pressure of results, professional and personal environment, can increase the risk of injury in a high-level athlete (40, 41). Also, the recovery due to energy expenses, specific to high performance sport, is essential. The quality and the quantity of recuperation strategies (e.g., sleep, nutrition, hydrotherapy, etc.) to optimize the high-level athlete performance showed benefits (42, 43).

This narrative review was conducted using a structured but non-systematic search strategy aimed at identifying conceptual, theoretical, and empirical literature relevant to motor development, neuroplasticity, motor control, and elite athletic performance. Searches were performed in PubMed, Scopus, Web of Science, and Google Scholar databases using combinations of the following keywords: motor development, motor milestones, neuroplasticity, motor learning, proprioception, coordination, warm-up, injury prevention, elite athletes, and performance optimization.

Studies were included if they (i) addressed neuro-sensorimotor mechanisms, (ii) examined training or rehabilitation approaches affecting motor function, or (iii) contributed theoretical frameworks relevant to movement organization. No restriction on publication date was imposed because foundational developmental theories remain conceptually essential.

The selection of 57 references reflects purposive sampling typical of narrative reviews, prioritizing conceptual relevance, methodological rigor, and representativeness of key scientific domains rather than exhaustive coverage. This approach is consistent with the hypothesis-generating objective of the present work.

As EPAs, especially in team sports, are always asking for specific training proposals to improve biomechanical, physiological and psychological requirements, the hypotheses based on the MDS are presented below.

## 2 The hypothesis

The present hypothesis is grounded on three assumptions: (a) motor development stages reflect fundamental neural organization principles, (b) integrated multi-segment movements stimulate broader sensorimotor networks than isolated exercises, and (c) exposure to novel coordinated tasks may enhance motor adaptability.

In the present framework, a SCM is defined as a structured series of coordinated postures, movements and transitions derived from MDS and intentionally recombined into task-specific sequences. Each SCM is characterized by four parameters: (i) selection of developmental movement components, (ii) sequencing order, (iii) execution speed (slow, moderate, fast), and (iv) task-specific adaptation to sport demands.

Unlike conventional neuromuscular or proprioceptive drills, SCM emphasizes multi-positional transitions, whole-body coordination, and progressive sensorimotor integration rather than isolated muscle activation or single-skill rehearsal. SCM therefore constitutes a combinatorial motor framework rather than a single exercise modality.

It is important to clarify that the present article does not assume a direct equivalence between neurological rehabilitation protocols and elite athletic training. Instead, it proposes that both domains may share underlying neurophysiological principles related to motor learning, neural adaptation, and sensorimotor integration. Rehabilitation research provides a conceptual model illustrating how structured movement sequences can reorganize motor function. The current work explores whether analogous principles could be adapted—not transferred identically—to enhance performance capacities in already highly trained individuals.

Why and how should we apply combinations based on motor development stages MDS in a simple and effective way to the healthy, trained and elite athlete? We know that MDS can be applied to patients with neurological disabilities in a specific and adapted way, depending on each neurological disorder, sensory and motor damage. The purpose in these cases is to restore sensitive functions, movement and posture capacities, and therefore the functional faculties necessary for ADLs, in order to improve QoL.

The daily activity of EPAs also involves cognitive, sensory, motor, postural and functional capacities at low, medium and high intensity, in a defined and variable spatio-temporal context. The cerebral, cognitive and emotional, bodily and gestural requirements of the EPAs therefore require special attention. For us, it's not a question of repeating the MDS as described by Le Métayer. Rather, it's a matter of transposing the MDS to high-level athletes, by creating multiple combinations of postures and movements, and individualized sequences, which enable high-level athletes to benefit from it. For them, the objectives, in the short, medium and long term, are to (i) prevent injuries or rehabilitate movement after an injury with a view to resuming activity, (ii) improve brain capacities for precise execution gestures in low, medium and high intensity, and for body awareness. Indeed, MDS reproduce the neuro-sensory-motor bases of motor development observed by Le Métayer, in newborns, from birth to the first steps of walking. Each individual accomplishes the different stages of the MDS, in a complete or incomplete way. For the patient suffering from neurological disorders, it is therefore a question of reproducing the different phases of the MDS, by a principle of “reprogramming” (3) and efficiency of his own neuro-sensory-motor system with a view to the recovery or even the cure of his pathology. In rehabilitation, the repetition of motor tests, the presence of subjective and objective feedback, as well as knowledge of spatio-temporal modalities and intensity, allow the patient to better “self-organize”.

The premise of our approach recognizes the 17 steps as they were described by Le Métayer (Table 1). However, what makes our methodology unique is the combinations of different movements that make up each sequence. We are talking here about specific SCM that will combine some of the stages of motor development that occur from birth to standing for walking. The possibilities of combining the 17 steps are multiple SCM, adaptable to each sport, or to each EPAs. Additionally, we utilize three different speeds, including slow, medium, and fast paces. The slow pace aims to facilitate comprehension, learning, integration, and memorization of the sequences. The medium pace allows for an increase in the execution velocity as a warm-up routine. Finally, the fast pace is similar to the speed of the gesture at very high intensity, which is specific to the training and competition. SCM are structured progressions of whole-body postural transitions derived from MDS, performed at controlled tempos to simultaneously challenge coordination, balance, proprioception, and cognitive-motor integration.

Even elite athletes, despite their advanced motor skills, may benefit from revisiting earlier stages of MDS. Such regressions can reinforce foundational movement patterns, including balance, coordination, and joint stability, which support movement efficiency and adaptability. By re-engaging these basic patterns, athletes may correct subtle inefficiencies, enhance coordination variability, and reduce injury risk, although these effects require empirical validation.

For the purposes of this review, we will enounce only two sequences of combinations of movements (SCM1 and SCM2 illustrated in Tables 3, 4), which were adapted from the MDS.

**Table 3:** The first combination of positions and movements from the MDS (SCM1).

First combination of positions and movements (SCM1)
1. Supine
3. Turnovers: back to stomach
2. Prone position: position of the sphinx
12. All fours position, ready to crawl in all fours position
5. Rabbit position (kneeling): sitting on its buttocks
6. On his knees erect: without support of the hands
9. Position of the serving knight
13. Passage to a standing position: from the position of the serving knight
16. Standing without support
17. Walk: forward and followed by a half turn then back to the initial position
1. Supine

**Table 4:** The second combination of positions and movements from the MDS (SCM2).

Second combination of positions and movements (SCM2)
1. Supine
3. Turnovers: back to sideways
7. Sitting beach position (position of the little mermaid (one side)
7bis. Sitting beach position (position of the little mermaid (other side)
9. Position of the serving knight
13. Passage to a standing position: from the position of the serving knight
16. Standing without support
17. Walk: forward and followed by a half turn then back to the initial position
1. Supine

The two sequences presented here (SCM1 and SCM2) were selected as illustrative examples based on specific construction principles: progressive movement complexity, inclusion of transitional postures, bilateral symmetry, feasibility within training contexts, and potential transferability across sports. They are intended to demonstrate the conceptual logic of SCM design rather than represent exhaustive protocols.

The parallel between the application of the MDS and the SCM is therefore conceivable for the EPAs, with regard to the questions of (i) effectiveness of the warm-up routines, (ii) post-training and competition recovery, (iii) injury prevention and post-injury rehabilitation for return to training, and (iv) brain, movement, gesture and body capacities. These problems are often related to the time factor, to the will, even to the almost permanent need to shorten recovery and healing times. Therefore, relapses following an injury are recurrent. For these reasons, SCM would have positive effects on injury prevention, rehabilitation, optimization of brain functions, and on the performance of movement, gestures and sports technique. Mastering these brain and body skills, through the practice of SCM, would help and encourage EPAs to better understand and adapt to the constraints of their personal, professional and sporting lives, particularly during repeated elite and high-level training sessions and competitions.

## 3 Evaluation of the hypothesis

The application of a SCM adapted from the motor development stages could produce beneficial effects in EPAs. Primary dimensions such as movement, posture, coordination, balance, joint mobility, are related to brain functions and structures and to specific brain mechanisms, including neuroplasticity (44). Learning a new motor skill, along with the experience and ability to perform different combinations of movements, always expands a person's motor repertoire (45, 46). It requires substantial brain activation, due the new processing of input and output information concerning attention, sensations, perception, decision making and memory. Therefore, the execution of a new movement stimulates distinct brain areas. Furthermore, persistent learning of these novel motor tasks expands the individuals’ motor repertoire and promotes gray matter plasticity (46, 47). Secondary benefits are obtained on the cardiorespiratory (ventilation, heart rate, heart rate variability) and psychological (emotional states, stress levels) systems (48). On the other hand, we know that heartbeat perception is considered to be a capacity linked to interoception which is the detection and perception of stimuli originating from inside the body (49). Thus, sprinters and distance runners were recently described to be more confident than non-athletes in their cardioception (50). All of these effects mentioned above could improve QoL and ADLs.

One of the potential benefits of SCM for high-level athletes lies in joint mobility, due to the gain in joint amplitude obtained by SCM movements and by the positioning of the bone segments which favor the opening of the angles of the hinges of the movement. Muscle tone is also regulated by the SCM, which would help the athlete's body to adapt during training and competition. Furthermore, the elasticity of the muscle, commonly obtained by stretching techniques (51, 52), is an element that could be acquired by the SCM, which create a different type of elongation of the muscle, in the active and voluntary phase of movement. Furthermore, it is important to consider the incorporation of delicate input in order to enhance perception and proprioception (39). The execution of the SCM serves to activate both internal and external proprioception, which is crucial for precise movement and excellent athletic technique displayed by the EPAs. The various speeds utilized during the SCM, including slow, medium, and fast paces, significantly challenge motor control, as it is a key factor in performance.

SCM should therefore be interpreted as active task-oriented neuromotor engagement rather than a substitute for stretching interventions.

With respect to SCM, its beneficial effects would be that they represent targeted training of the upper torso and core stability, which would benefit athletic performance and perhaps provide a better understanding of how trunk strength and stability influence overall athletic performance. Indeed, the systematic review by Reed's team (53) defines upper torso and core stability training as training focused on improving trunk and hip control as an integral part of athletic development. Their review raises the question between core stability and athletic performances, and identifies the difficulties encountered when trying to train core stability in order to improve athletic performance.

The benefits of SCM on functional aspects (position changes, transfers, ground perception, walking) of patients with neurological disabilities (3, 11) would also be valid for EPAs; we could obtain improvements on running speed, on jumps, on changes of direction, on U-turns. These functions driven by body warm-ups and movement in space, would be enhanced by the SCM applied, as a variety of warm-up, or even a pre-warm-up. We can only assume that the reconfiguring and reprogramming nature of functionality by SCM, would act as an injury prevention factor, due to the improved quality of gestures and spatio-temporal body movements.

In addition, the SCM are learned and improved by repeating the motor task. With regard to learning, a recent study has provided information on the interest of application of internal and external visual imagery, in the sports field in particular, notably Pilates (54). Authors demonstrated that visuomotor imagery (VMI) is not useful for individuals who lack specific motor experience. They analyzed the time of execution, and the imagination, that is to say the visualization of a sequence of movements in the first-person perspective (internal VMI-I) and in third person (external VMI-E) for two types of Pilates exercises, in individuals with different skill levels (expert, novice and non-practicing individuals). The results showed that in expert individuals, in the VMI-I perspective, the imagination time was similar to the execution time, while in the VMI-E perspective, the imagination time was significantly less than the time of execution. An opposite pattern was found in the novice group, in which the imagination time was similar to that of execution, only in the VMI-E perspective, while in the VMI-I, the imagination time was significantly less, at runtime. Authors postulated that while VMI-I is used to train an already internalized gesture, the VMI-E perspective may be useful for learning, and then improving, the recently acquired sequence of movements. The SCM could thus help synchronize the visualization and execution of a specific movement for an expert athlete. This is also supported by the study of Guillot's team (55) which demonstrated that dynamic imaging could contribute to improve the quality and efficiency of motor imagery in high jumpers, leading to potentially fruitful new directions for sports practice.

In addition, the posture and innate movement patterns, balance and coordination are factors inherent in the technical success of the sporting gesture. It has been reviewed that the most successful athletes in term of sport competition level have the best postural performance both in ecological and non-ecological postural conditions (56). Also, described as limiting elements, in case of disorders thereof, the team of Sarto and al (57), demonstrated in a laboratory protocol that endurance and team players showed different behavior in coping with static and dynamic balance both in single- and dual-task conditions. These observations should be considered when researching and developing new training techniques.

The biomechanical and cerebral contribution of the SCM, through the application of innate basic movements, of a pattern of repeated, symmetrical movements, eyes open and eyes closed, brings possibilities of performance of the body and of the gesture whatever the sport and its spatial, temporal and gestural solicitations (1, 2). Moreover, just like in the life of patients with neurological disorders, the guarantee of optimal ADLs and a preserved QoL, is essential in the life of EPAs. Thus, training in SCM would also be useful, to improve ADLs and QoL.

The appropriate use of SCM and a combination of neuro-motor rehabilitation methods in EPAs could increase body awareness, prevent and manage injuries, aid in post-rehabilitation, improve warm-up routines and prepare for training and competition, and improve brain performances.

## 4 Limitations

Several limitations must be considered. First, this review is narrative and hypothesis-driven and therefore does not provide experimental validation of the SCM efficacy. Second, the proposed sequences (SCM1 and SCM2) originate partly from professional expertise and should be considered preliminary models rather than standardized protocols. Third, extrapolation from rehabilitation populations to elite athletes may not be directly generalizable because these populations differ substantially in baseline motor capacities, neural efficiency, and training history. Finally, although theoretical mechanisms are biologically plausible, their magnitude of effect in high-performance sport remains unknown and requires controlled longitudinal studies.

## 5 Perspectives

Evidence cited in this review ranges from clinical rehabilitation trials to theoretical neuroscience models. Direct evidence in elite athletes remains limited and extrapolations should therefore be interpreted as hypotheses requiring experimental validation. With regard to our professional expertise in elite sport and supported by scientific literature, we posit on the interest of further research to investigate the mechanisms and responses of the brain and body when using SCM in healthy subjects and EPAs. We will conduct laboratory studies to validate the effects of SCM in the short, medium, and long term. In the acute phase, we will study its immediate effects on physical, physiological, and cognitive skills, such as mobility, coordination, balance, and cardio-respiratory capacity. In the chronic phase, we will study its longer-term effects on measures of performance, such as neuroplasticity. We will also assess the potential for reeducation, quick and efficient return to training and competition, warm-up routines, and injury prevention. Experiments will be conducted in individual and elite team sports.

Methodological challenges include standardizing SCM protocols, adapting sequences to sport-specific constraints, ensuring feasibility within elite training schedules, and isolating SCM-specific effects from general training load. A legitimate counterargument is that elite athletes may already exhibit optimized motor patterns and therefore may not benefit from revisiting developmental movement sequences. It is also possible that regression toward earlier motor patterns may be unnecessary once advanced sport-specific automatisms are established. Another alternative explanation is that any benefit attributed to SCM could simply reflect general effects of novel coordination training rather than the specific structure of developmental-based sequences. These competing interpretations highlight the necessity of controlled comparative studies designed to isolate SCM-specific effects.

Taken together, SCM should be viewed as a conceptual training paradigm grounded in developmental motor principles that warrants empirical investigation. Its scientific value lies not in established efficacy but in its potential to generate testable hypotheses at the intersection of motor development, neuroscience, and elite performance science.
